# Nonvolatile photonic field-programmable coupler array

**DOI:** 10.1126/sciadv.aec7329

**Published:** 2026-05-01

**Authors:** Håvard Hem Toftevaag, Bowei Dong, Nikolaos Farmakidis, Angel Ortega-Gomez, Harish Bhaskaran

**Affiliations:** ^1^ Department of Materials, University of Oxford, Parks Road, Oxford OX1 3PH, UK.; ^2^ Institute of Microelectronics (IME), Agency for Science, Technology and Research (A*STAR), 2 Fusionopolis Way, Innovis #08-02, Singapore 138634, Singapore.

## Abstract

Programmable photonic networks carry out universal unitary functions by independently operating on the amplitude and phase of guided light. Exploiting the reconfigurability and spatiospectral degrees of freedom of these systems, the majority of state-of-the-art photonics applications, ranging from microwave photonics to photonic computing and optical communication links, can be demonstrated in one unified system. Existing techniques require a large footprint due to weak modulation efficiency, and continuous power dissipation to maintain the configured state. Here, we demonstrate a programmable recirculating mesh unit cell based on the nonvolatile low-loss phase-change material Sb_2_Se_3_. The demonstrated devices achieve an ultrashort active length (<10 μm, more than 15 times smaller than the current state of the art of competing technologies) and zero static power, in combination with high-extinction switching (>20 dB), broadband operation (>15 nm), and low insertion loss (<2 dB). This work forms the basis for nonvolatile field-programmable coupler arrays (nv-FPCAs) and zero–static power reconfigurable optical interconnects.

## Teaser

Nonvolatile phase-change photonics enable ultracompact, zero–static power programmable networks for scalable photonic computing.

## INTRODUCTION

Manipulating the electromagnetic spectrum within on-chip components is central to enabling technologies in microwave photonics for radio frequency (rf) and radar systems (*1*, *2*), optical signal processing (*3*), computer hardware accelerators (*4*, *5*), classical (*6*) and quantum computing (*7*), and, crucially, optical interconnects (*8*). Photonic integrated circuits (PICs) remain, in most cases, application-specific, meaning that they are designed to carry out a single operation efficiently but lack postfabrication reconfigurability (*9*–*11*). Several pioneering works in integrated photonics have recently demonstrated dynamic reconfiguration using transient electrical signals through thermo-optic, electro-optic, piezo, and optomechanical effects (*9*, *12*–*20*). While these systems greatly enhance the reconfigurability of PICs, they require unsustainably large power budgets to maintain each configuration and long modulator lengths, which leads to large footprints and limits the spectral characteristics of the synthesized components.

In space- and power-limited systems, such as edge sensors, CubeSats, and microdrones, the requirements are even more challenging. Under such extreme conditions, the ideal photonic chip should not only have a reasonably small footprint, but also be fine-tunable and near-lossless, as well as accommodate wavelength-division multiplexing. The individual devices should ideally have short stabilization times and low standby power. At a system level, the electrical driving signals should occur within complementary metal-oxide semiconductor (CMOS)–compatible voltage ranges, with minimal thermal and electrical cross-talk between components.

**Fig. 1.**
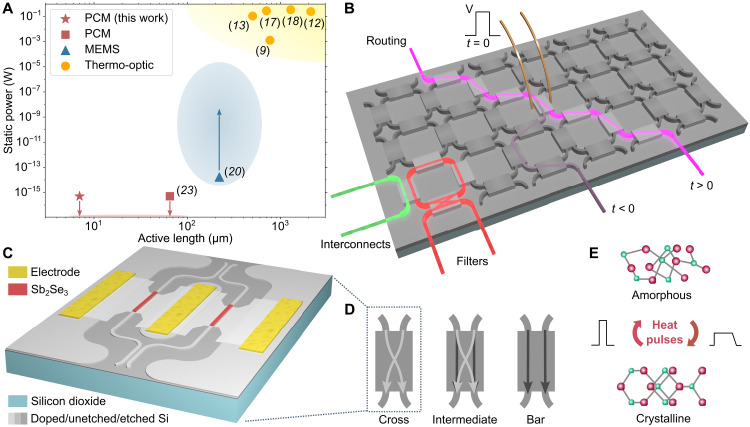
FPCA with Sb_2_Se_3_-based MZIs. (**A**) Static power consumption versus active length of the building blocks of demonstrated FPCA mesh technologies. For the microelectromechanical systems (MEMS) devices, the power consumption of electronic control circuits (such as DACs) substantially exceeds the power of the devices themselves, meaning the actual static power consumption is substantially higher, as indicated by the blue arrow. (**B**) Conceptual illustration of an FPCA mesh with different optical paths that can be used for routing, interconnects, filters, and more. An electrical pulse (V) is sent at *t* = 0, changing the path of the pink light. (**C**) Schematic of the tunable coupler in this work: a balanced MZI with the PCM Sb_2_Se_3_ as the phase shifter in each arm of the MZI. The capping layer of ZnS-SiO_2_ is not shown here. (**D**) Schematics showing the different states of the tunable couplers in the FPCA mesh: the cross, intermediate, and bar states. (**E**) Schematic showing the Sb_2_Se_3_ switching between the amorphous (unordered) and crystalline (ordered) state with different heat pulses.

Here, we demonstrate a compact, zero–static power, nonvolatile field-programmable coupler array (nv-FPCA), which combines key PIC components (waveguides, couplers, and interferometers) with emerging low-loss active materials. Central to this system is the recently discovered optical low-loss phase-change material (PCM) Sb_2_Se_3_ (*21*–*24*), which shows a strong, stable, and reversible modulation in its refractive index when switched between the two material states (Δ*n* > 0.64) with ultralow extinction coefficient (*k* < 0.02) in the telecoms C band. In the nv-FPCA, Sb_2_Se_3_ acts as the phase shifter of the tiled 2 × 2 Mach-Zehnder interferometer (MZI) couplers making up the mesh. The tunable couplers show high–extinction ratio (ER) switching (20 dB) over a wide bandwidth (15 nm), with an insertion loss of 1.4 to 1.8 dB and reconfiguration energy of 1.2 μJ and 1.0 mJ for the two extreme states of the coupler, respectively. Due to the nonvolatile behavior of the PCM, our system has no static power consumption and needs no electrical driving once the configuration is set. Furthermore, the large refractive index contrast between the states of the PCM considerably reduces the coupler length to the order of tens of micrometers, facilitating compact reconfigurable resonators with larger free-spectral ranges (FSRs) required for optical signal processing applications (*12*). To showcase the general applicability of our nv-FPCA, we reconfigure it for three key components for PICs, namely, (i) routing of light, (ii) configuration as microring resonators, and (iii) synthesis of delay lines. Compared with other existing FPCA technologies presented in Fig. 1A, our nv-FPCA has an order-of-magnitude improvement in the active length of the building blocks and no static power consumption.

## RESULTS

### Working principle

A three-dimensional schematic of an FPCA mesh is shown in Fig. 1B, with the tiled 2 × 2 tunable coupler in Fig. 1C forming the square recirculating mesh. Each tunable coupler can be set to either the cross state (100% coupling across the MZI), the bar/through state (0% coupling across), or intermediate states providing variable splitting of the light (Fig. 1D). The tunable couplers in this work are 2 × 2 balanced MZIs with 50/50 directional couplers at the input and output, fabricated on a CMOS-compatible silicon-on-insulator (SOI) platform, with the low-loss PCM Sb_2_Se_3_ acting as a nonvolatile phase shifter coupled to each arm of the MZI in the nearfield. We set the state of the MZI coupler by changing the optical phase difference, Δφ, between the two PCM elements, where Δφ = 0 gives the cross state and Δφ = ±π gives the bar state. This phase difference arises from the large change in refractive index between the crystalline and amorphous states of the Sb_2_Se_3_ (Fig. 1E), $\Delta n_{Sb_{2}Se_{3}}=n_{\text{cry}}-n_{\text{amo}}\approx 0.65$ (full ellipsometry data in text S1), which leads to a change in the effective refractive index of the PCM-covered waveguide (see text S2).

**Fig. 2.**
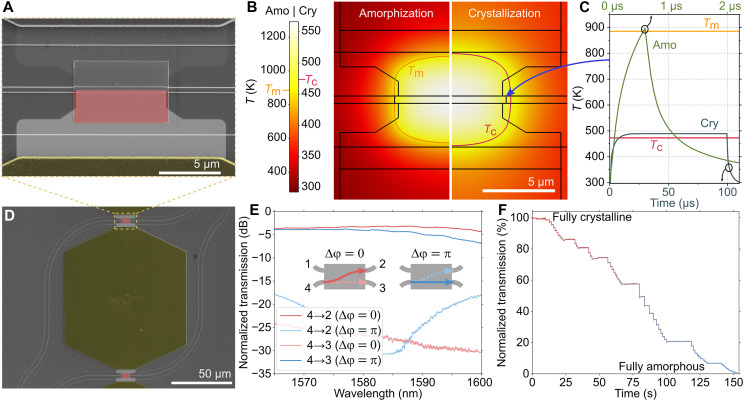
Phase shifter and tunable coupler with Sb_2_Se_3_. (**A**) Scanning electron micrograph of the phase shifter, false-colored only in the bottom half, showing the electrodes (gold-colored) and boron-implanted microheaters (light gray) with Sb_2_Se_3_ and a capping layer of ZnS-SiO_2_ (red) on top. (**B**) Finite element method (FEM) simulation of the microheater temperature under electrical pulse excitation for amorphization (Amo) and crystallization (Cry) pulses. (**C**) Transient temperature at the top corner of the PCM patch (indicated by the arrow). The temperature reaches the melting point *T*_m_ = 885 K and crystallization temperature *T*_c_ = 473 K for the amorphous and crystalline pulses, respectively. (**D**) False-colored scanning electron micrograph showing two phase shifters in an MZI coupler. (**E**) Normalized transmission spectrum of an individual MZI in the cross state (Δφ = 0) and bar state (Δφ = π) with light entering port p4. (**F**) Normalized transmission from the cross port of an MZI coupler under sequential amorphization of one of the phase shifters, with both phase shifters initially in the crystalline state, showing >50 distinct levels.

To achieve programmability, we engineer microheaters directly in the silicon through boron implantation; this approach allows us to electrically set the state of the Sb_2_Se_3_ via joule heating in the microheaters. Aluminum (Al) pads were deposited on both sides of the microheater, forming ohmic contacts with the doped silicon (see Methods and text S3). The bow-tie shape of the heater (Fig. 2A, with detailed parameters in fig. S4) is designed to effectively allow local joule heating in the area around the PCM without causing thermal cross-talk with neighboring devices (Fig. 2B) (*25*). A trade-off exists between the doping concentration, which affects the mode loss, the size of the active region, and the modulation strength achievable. The demonstrated devices were implanted with a dose of 3 × 10^15^ ions/cm^2^ at an energy of 10 keV, which was measured to induce an optical loss of 0.11 dB/μm (fig. S5).

Optical measurements of waveguides covered with Sb_2_Se_3_ are presented in text S6. The change in the effective refractive index is Δ*n*_eff_ = 0.1 for a 60-nm-thick film, which means that the length of the PCM element needs to be *L*_PCM_ = 7.6 μm to achieve a full π shift, in good agreement with simulations (text S2). Furthermore, the experimental loss of the thin film was 0.1 dB/μm for the most lossy (crystalline) state, giving a 0.8-dB loss for a π shift. These numbers are reduced to 0.02 dB/μm and 0.3 dB/π for a 30-nm-thick and 16.1-μm-long Sb_2_Se_3_ patch. However, because the total loss of the phase shifter includes the non-negligible loss of the integrated heaters, we used 60-nm-thick Sb_2_Se_3_ to reduce the total loss. Furthermore, we limited the length of the microheaters to 7 μm to minimize the total heater loss while still reaching temperatures sufficient for full π phase shift (Fig. 2, B and C). We deposited a 60-nm-thick and 7.6-μm-long patch of Sb_2_Se_3_ on top of the central part of the heater in a lift-off process using rf magnetron sputtering, with a 20-nm-thick capping layer of ZnS-SiO_2_ to impede oxidation and delamination of the PCM. For more details on the fabrication, see Methods and text S7.

### Tunable coupler

First, we characterize the building block of the FPCA, which in this work is an individual MZI coupler with two Sb_2_Se_3_ phase shifters, shown in Fig. 2D. We show optical measurements of an individual MZI coupler in Fig. 2E, where the tunable coupler is set to the cross state (Δφ = 0) and bar state (Δφ = π). The spectra show low cross-talk between the ports, with a maximum ER at wavelength λ = 1582.5 nm of ER_bar_ = 22.6 dB and ER_cross_ > 27.6 dB (limited by the photodetector’s noise floor), with an ER of at least 20 dB over 15 nm. The spectra in this work are normalized to the transmission of a plain waveguide, to account for the Gaussian-like transmission spectrum of the grating couplers. In text S8, we estimate the loss of the tunable MZI coupler to be in the range of 1.4 to 1.8 dB per coupler.

The tunable couplers in this work can also reach several intermediate levels between the extreme states. In Fig. 2F, we set the transmission of a tunable coupler to >50 distinct transmission levels by sending amorphization pulses of varying energy to the phase shifter in one arm, with pulse parameters found in table S3. In fig. S8, we also show repeated switching of Sb_2_Se_3_, although with a limited ~0.13π phase shift. After complete amorphization, we were only able to crystallize ~50% of the PCM volume, as seen in fig. S8A. This limitation might be due to poor thermal engineering, material degradation or segregation, or partial delamination of the PCM caused by the high-temperature amorphization pulses. As outlined in text S9, the total energy used to achieve a full π phase shift by amorphization was 1.2 μJ, and we estimate ~1.2 mJ for crystallization. These numbers are in part caused by the pulse sequences used, which we used to ensure device integrity throughout the experiments. For optimized pulse parameters, we expect these numbers to decrease.

### FPCA as an optical router

**Fig. 3.**
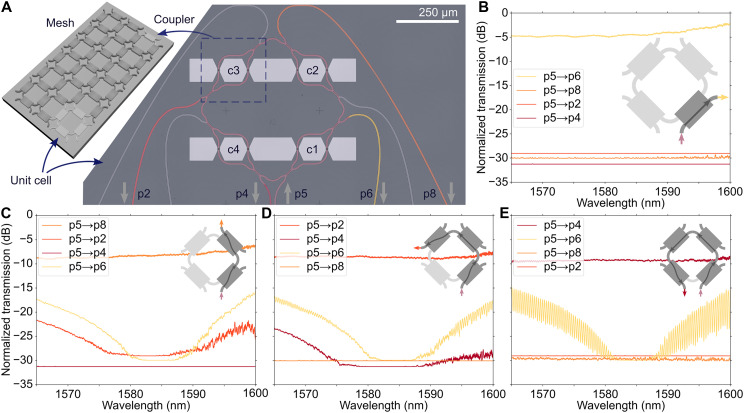
Routing of light out of different output ports of the FPCA. (**A**) False-colored optical micrograph of the fabricated FPCA unit cell. The inset shows the relation between an FPCA mesh, a single tunable coupler, and the fabricated unit cell. (**B** to **E**) Normalized transmission spectra from the different output ports of the FPCA unit cell, for when the c1 coupler is in the bar state (B); c1 and c2 are in the cross state (C); c1 in cross, c2 in bar, and c3 in cross state (D); and, last, c1 in cross, c2 and c3 in bar, and c4 in cross state (E). The insets in each figure show the configuration for each measurement.

We proceed to show three different optical functions based on the reconfigurable mesh: routing, synthesis of a resonator, and synthesis of a delay line. Figure 3A shows an FPCA mesh and the unit cell presented in this paper, which consists of four tunable MZI couplers in a square lattice arrangement. First, we demonstrated routing of light out of the different output ports of the device according to the schematics in Fig. 3 (B to E). In these figures, light is sent into port p5, and the FPCA is configured to route light out of port p6, p8, p2, and p4, respectively. The corresponding configurations of the FPCA are coupler c1 in the bar state (p5 → p6 has the highest output); c1 and c2 in the cross state (p5 → p8); c1 in cross, c2 in bar, and c3 in the cross state (p5 → p2); and last, c1 in cross, c2 and c3 in bar, and c4 in the cross state (p5 → p4). The FPCA shows broadband behavior, effectively routing light over a wide range of wavelengths. The ERs between the different output ports when the FPCA unit cell is in the configurations in Fig. 3 (B to E) are >20 dB for wavelengths between λ = 1581 and 1588 nm.

### FPCA as a ring resonator

**Fig. 4.**
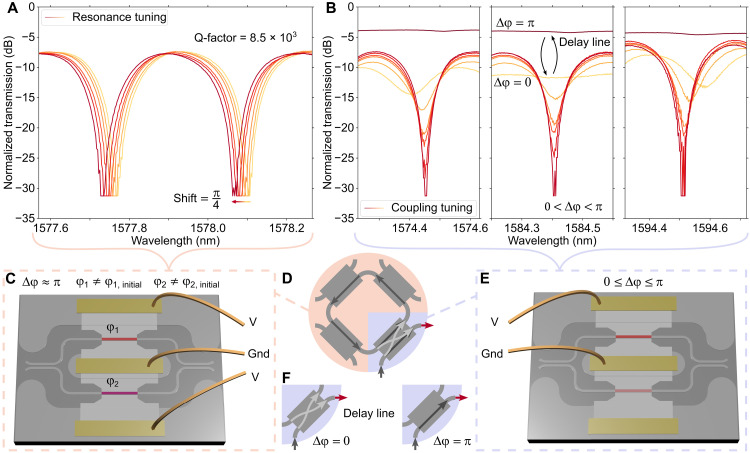
The FPCA as a ring resonator and delay line. (**A**) Normalized transmission spectra showing tuning of the resonance wavelength of the synthesized ring resonator near critical coupling. Tuning is achieved by changing the effective refractive index of the ring with the phase shifters in the couplers in the ring [peach-colored in (D)]. (**B**) Normalized transmission spectra of the ring resonator at different states; with the input tunable coupler in the bar state (Δφ = π), in the cross state (Δφ = 0), and in several intermediate states (0 < Δφ < π), including close to critical coupling (sharpest resonance). In (A) and (B), the colors represent the sequential tuning from the initial state (yellow) to the final state of the measurements (dark red) with several intermediate states. (**C**) Schematic showing the coupling conditions for the couplers in the ring, for the transmission spectra in (A). (**D**) Schematic of the synthesized ring structure. (**E**) Schematic showing the coupling conditions for the input coupler, for the transmission spectra in (B). (**F**) Schematics showing how the FPCA unit cell can be used as a reconfigurable delay line by setting the input coupler to the extreme states. Gnd, ground electrode.

To further demonstrate the potential of our FPCA unit cell, we configured it to synthesize a ring resonator, i.e., when three of the tunable couplers guide light around the unit cell and the fourth is set to an intermediate state governing the coupling into the ring. In Fig. 4, we show transmission spectra for the synthesized ring resonator when the cross-coupling coefficient is close to 0 (bar state) and 1 (cross state), as well as several intermediate states, including close to critical coupling. In Fig. 4 (A and B), the colors represent the sequential tuning from the initial state (yellow) to the final state of the measurements (dark red) with several intermediate states. As seen in Fig. 4A, we can shift the resonance of the ring by simultaneously changing the PCMs of both arms of the guiding tunable couplers, i.e., changing φ in both arms of the tunable coupler but maintaining Δφ between the arms (as described in Fig. 4C). We can achieve larger resonance shifts by depositing longer PCM patches.

Next, we show that both the resonance wavelength and transmission are changed when we switch the intermediate-state MZI used for in-coupling, as shown in Fig. 4B. Figure 4 (A and B) is similar to the figures in the work by Lian *et al.* (*26*), where the authors use a single PCM element instead of MZIs for nonvolatile phase modulation and coupling (at the drop port) of a ring resonator. We find the FSR and quality factor (Q-factor) of the synthesized ring by fitting the troughs in the spectra to Lorentzian functions, as presented in text S10. The synthesized ring resonator at near critical coupling has a loaded Q-factor of 8.5 × 10^3^, an FSR of 0.33 nm (40 GHz), and an ER_ring_ of >23.7 dB for the resonances shown in Fig. 4A.

### FPCA as a reconfigurable delay line

Last, we show that we can synthesize a reconfigurable delay line with the FPCA. With the input tunable coupler in the bar state (cross-coupling set to 0), the light does not couple to the ring (marked with Δφ = π in Fig. 4, B and F). However, with the coupler in the cross state (coupling set to 1), almost all the light goes into, around, and out of the ring without interfering with itself (marked with Δφ = 0 in Fig. 4, B and F). By going between these two extreme states, the FPCA effectively acts as a nonresonant delay line with a group delay of 28 ps, which can be increased if we add more unit cells. As can be seen in Fig. 4B, this is only true for wavelengths around 1584 nm, where the directional couplers at the input and output of the MZI has 50% splitting, and the MZI therefore shows maximal extinction. With the input coupler in the cross state, the ER of the resonance at 1584.4 nm is only 0.6 dB, which is validated by simulations in text S10.

## DISCUSSION

We have demonstrated an nv-FPCA recirculating mesh unit cell with the low-loss PCM Sb_2_Se_3_ as a nonvolatile phase shifter, which allows for zero–static power and small-footprint building blocks for the mesh. In this work, we demonstrated how the nv-FPCA can be used for optical interconnects and to synthesize a ring resonator and a reconfigurable delay line. By scaling the system, we can achieve more complex circuits, such as longer delay lines, synthesized MZIs, and higher-order ring resonator filters (*12*, *13*). Table 1 summarizes the main parameters of this and other FPCA demonstrations and compares their performance. In addition, we show the performance of a future PCM-based device with thinner Sb_2_Se_3_ of 30 nm and p-i-n junctions for reduced overall loss. Notably, the PCM-based couplers in the nv-FPCA feature substantially shorter active lengths than those in other implementations and are nonvolatile, removing the need for continuous electrical driving from relatively power-hungry voltage sources.

**Table 1.** Performance comparisons of demonstrated FPCA technologies. Boldface indicates the best current and future result in each column. NA, not applicable; DC, directional coupler.

Technology	Power (*P*_π_)^*^	Switching energy	Loss (dB)	Unit length	Active length	Nonvolatile	Ref.
Thermo-optic MZI (SiN)	*P*_avg_ = 0.25 W	NA	NA (≲1)	3.45 mm	2.1 mm	No	(*12*)
Thermo-optic MZI (Si)	110 ± 15 mW	NA	0.6 ± 0.1	≥0.98 mm	~0.5 mm	No	(*13*)
Thermo-optic MZI (Si)	1.3 mW	NA	0.48	0.81 mm	767 μm	No	(*9*)
Thermo-optic DC (SiN)	0.35 W	NA	**0.2**	~2.1 mm	1.28 mm	No	(*17*)
Thermo-optic MZI (SiN)	0.290 W	NA	2	1.3 mm	~0.7 mm	No	(*18*)
MEMS (Si)	*P*_max_ < 17 fW	**<44 pJ**	0.26–0.52^†^	**0.38 mm**	150 + 100 μm	No	(*20*)
PCM-based MZI (Si)	**0**	NA^‡^	NA^‡^	~1 mm	77.8 μm	**Yes**	(*23*)
PCM-based MZI (Si)	**0**	1.2 μJ/~1.0 mJ	1.4–1.8	0.48 mm^§^	**7.6 μm**	**Yes**	This work
PCM-based (future)	**0**	<108 nJ/0.53 μJ^¶^	~0.5–1.0	**<0.1 mm**	<30 μm^#^	**Yes**	This work

* Power is given as the electrical power required to tune the couplers from 0 to 100% coupling (*P*_π_), unless otherwise stated: *P*_avg_ is the average power consumption per heater, and *P*_max_ is the maximum static standby power consumption.

† Loss range, assuming one phase shifter per unit cell.

‡ Not enough information is presented to estimate the values.

§ Affected by the size of the metal pads.

¶ Values taken from Yang *et al.* (*24*).

# The length of the PCM element and heater could be made shorter, but at the expense of smaller achievable phase tuning (done in this work).

The tunable couplers demonstrated are constrained by fabrication limitations in our system and could be miniaturized even further. We show in text S11 that the optical path length can be reduced to ~0.1 mm per tunable coupler by using electrical vias instead of the large electrical pads required in our measurement setup. Furthermore, the switching energies of our devices are about an order of magnitude greater than those reported by Ríos *et al.* and Yang *et al.* for similar devices (*22*, *24*). The higher energy required mainly stems from using repeated pulses and can be reduced in improved devices with better thermal engineering for uniform heating and optimized pulse sequences.

Low-loss PCMs offer an interesting complementary alternative to the existing FPCA technologies shown in Table 1. PCMs allow for the smallest footprint of all optical materials platforms (*27*), which is crucial to improve the granularity of the FPCA mesh and to synthesize resonant structures with larger FSRs. PCMs also offer stable operation with long retention times (*28*), as well as wide-bandwidth devices due to the small wavelength dependence on Δn in the wavelength region of interest. Because each phase shifter needs not only PCM but also a heater, the insertion loss of even the most optimized devices is likely to remain at the higher end of the range compared with thermo-optic and microelectromechanical systems (MEMS) platforms, but still within acceptable limits. A related issue is the phase-dependent loss between the crystalline and amorphous states, which leads to power imbalance between the outputs of the MZI. A major limitation at the moment, and the focus of substantial ongoing research, is the material degradation [caused by, for instance, void formation or ablation; (*22*, *24*)] and limited cyclability of low-loss PCMs in optical devices. Solutions are already being explored, e.g., through thermal engineering (*22*) or using PCM subcells instead of one patch (*24*). We believe that as the technology matures, we will reach levels acceptable for infrequent switching applications such as FPCAs. The relatively high switching energies and long reconfiguration times of low-loss PCMs are not ideal, but are also not detrimental to FPCAs, where stable, nonvolatile couplers are preferred. Last, as the technology matures, we also expect to see true CMOS compatibility in terms of integration into foundries and lower switching voltages.

The total power consumption of the nv-FPCA building blocks depends on the number of switching events per second. Of the other technologies presented in Table 1, the MEMS devices are by far the most energy efficient for programming, with picojoule switching energy and femtowatt standby power dissipation. In comparison, PCM-based couplers have nano-to-millijoule switching energy but zero standby power consumption. With the conservative switching energies demonstrated by Ríos *et al.* (*22*), PCM-based heaters require on average 19.3 μJ per switching event; therefore, for PCM couplers to be more energy efficient than MEMS couplers, the switching needs to happen less than every 36 years. However, this assumes no electrical driving of the MEMS devices. In reality, the actual power consumption of the MEMS couplers will be dominated by driving electronics (*7*), such as the digital-to-analog converters (DACs) setting the state of the couplers [requiring, e.g., from nanowatts (*29*, *30*) to milliwatts (*31*–*33*)] and other control components, as indicated in Fig. 1A. Assuming total driving power consumption of 10 nW, the crossover point where PCM outperforms MEMS happens for switching once every 32 min, and already after less than 2 s for 10 μW. For applications where the setting of the circuit occurs infrequently, the advantage of nonvolatility becomes evident. Moreover, another benefit of nonvolatility is that we do not have to worry about reconfiguring the system in case of electrical driving failure. Therefore, we foresee the following use cases for the three different technologies: the reasonably low loss, large footprint, and large power consumption of the thermo-optic platform mean that it can be used for most prototyping and laboratory applications with relaxed size, weight, and power requirements; the MEMS platform is a great alternative that reduces the footprint and especially power and can find use in many implementations, such as for cryogenic environments; last, the PCM-based platform will perform optimally in space- and power-limited systems in extreme environments with infrequent FPCA switching requirements.

In summary, we have experimentally demonstrated a nonvolatile FPCA mesh unit cell with the low-loss PCM Sb_2_Se_3_ as the tuning element. Using the nv-FPCA unit cell, we demonstrate an optical router, a synthesized ring resonator, and a reconfigurable delay line, three key components for PICs. Both the unit cell and the individual building blocks show good performance in terms of ER and bandwidth when used for routing. The average reconfiguration energy of a single tunable coupler is ~0.55 mJ, but because Sb_2_Se_3_ is nonvolatile, there is no static power consumption and, thus, no requirement to maintain an electrical driving signal. The use of Sb_2_Se_3_ in our nv-FPCA will therefore greatly simplify the thermal design of the system, particularly with respect to thermo-optical tuning, as well as remove the need for constant DAC power consumption. Our demonstration paves the way for FPCAs with smaller footprint and zero static power consumption, for applications where size, weight, and power are crucial.

## METHODS

The FPCA unit cells were fabricated on an SOI wafer (Soitec SA) with 220-nm silicon on a 2-μm buried oxide layer. The rib waveguides and grating couplers were patterned in 400-nm-thick CSAR positive resist using a 50-kV electron-beam lithography (EBL) system (JEOL JBX-5500FS) and subsequently etched 120 nm using an inductively coupled plasma reactive-ion etching system (Oxford Instruments PlasmaPro 80 Cobra) with SF_6_ and CHF_3_ gases for silicon etching, followed by oxygen plasma for resist removal. The rib waveguide structure is seen in fig. S4. Windows for ion implantation were opened with EBL in a 3-μm-thick triple layer of polymethyl methacrylate (PMMA)–positive resist (one layer of 495 PMMA A8 and two of 950 PMMA A8) to protect the rest of the sample. Boron ions were then implanted with a dose of 3 × 10^15^ ions/cm^2^ at an energy of 10 keV. Following doping, the sample was immersed in acetone to remove the PMMA resist layer and then in piranha solution for 5 min to remove any resist residue or other organic residues.

Next, the samples were annealed at 920°C for 5 min in a tube furnace (Lenton Thermal Design LTF 12) under flow of argon to activate the boron dopant and alleviate the ion implantation damage. A 2-μm-thick double layer of positive resist (495 PMMA A8 and 950 PMMA A8) was then spin-coated and patterned using EBL to open windows for Al pads to contact the doped silicon in a lift-off process. The native oxide was removed in the openings using buffered oxide etchant to ensure ohmic contact between the pads and the doped silicon, before 300-nm Al was deposited by electron-beam evaporation. A final lithography step was done to deposit PCM on top of the heaters in a lift-off process. A double layer of positive resist (495 PMMA A8 and 950 PMMA A8) was patterned using EBL, and a stack of 60-nm Sb_2_Se_3_ and 20-nm ZnS-SiO_2_ was deposited using an rf-magnetron sputtering system (AJA International ATC ORION 5). The Sb_2_Se_3_ and ZnO-SiO_2_ targets were sputtered with a power of 30 and 50 W, respectively, with an argon flow of 3 SCCM (standard cubic centimeter per minute) at a base pressure of 3 × 10^−8^ torr. The process described here is very similar to that of the microheaters in our previous work on electrically programmable weight banks (*25*); the main difference lies in the annealing process and PCM deposition.

## Supplementary Materials

### This PDF file includes:

Supplementary Text Tables S1 to S5 Figs. S1 to S10 References
