# Supplementary material for: Nonvolatile photonic field-programmable coupler array
Source: Sci Adv. 2026 May 1;12(18):eaec7329. doi: 10.1126/sciadv.aec7329 (PMC13134612; doi:10.1126/sciadv.aec7329)
Supplement: Supplementary file 1 — Supplementary Text Tables S1 to S5 Figs. S1 to S10 References [file sciadv.aec7329_sm.pdf]

Science Advances
AAAS

Supplementary Materials for

Nonvolatile photonic field-programmable coupler array

Håvard Hem Toftevaag *et al.*

Corresponding author: Harish Bhaskaran, harish.bhaskaran@materials.ox.ac.uk

Sci. Adv. **12**, eaec7329 (2026)
DOI: 10.1126/sciadv.aec7329

This PDF file includes:

Supplementary Text
Tables S1 to S5
Figs. S1 to S10
References

Supplementary Text

S1 Ellipsometry of SbSe

S2 Mode simulations of SbSe-covered waveguide

S3 Electrical characterization of silicon-on-insulator microheater

S4 Detailed structural design parameters of phase shifter

S5 Optical loss due to ion implantation

S6 Optical measurements of SbSe on a rib-waveguide

S7 Fabrication steps

S8 Optical loss of SbSe-based tunable coupler

S9 Switching behaviour

S10 Synthesized ring resonator behaviour

S11 Future projections for footprint

S1 Ellipsometry of SbSe

Ellipsometry measurements of a 180 nm thick layer of Sb_2Se_3 (SbSe) were conducted using an J.A. Woollam RC2 ellipsometer and are presented in Fig. S1, where the data is fitted with two fitting models; Cody-Lorentz and Gaussian. As can be seen in Table S1, the refractive index difference between the crystalline and amorphous state of our SbSe, $\Delta n = n_{\text{cry}} - n_{\text{amo}}$, is about 0.65, with a wavelength dependency for Δn of only 0.014 between 1500 nm and 1600 nm.

Table S1: Overview of the real refractive indices of SbSe at different wavelengths.

Wavelength	n_{amo}	n_{cry}	Δn
1500 nm	3.3265	3.9838	0.6573
1550 nm	3.3172	3.9671	0.6499
1600 nm	3.3086	3.9522	0.6436

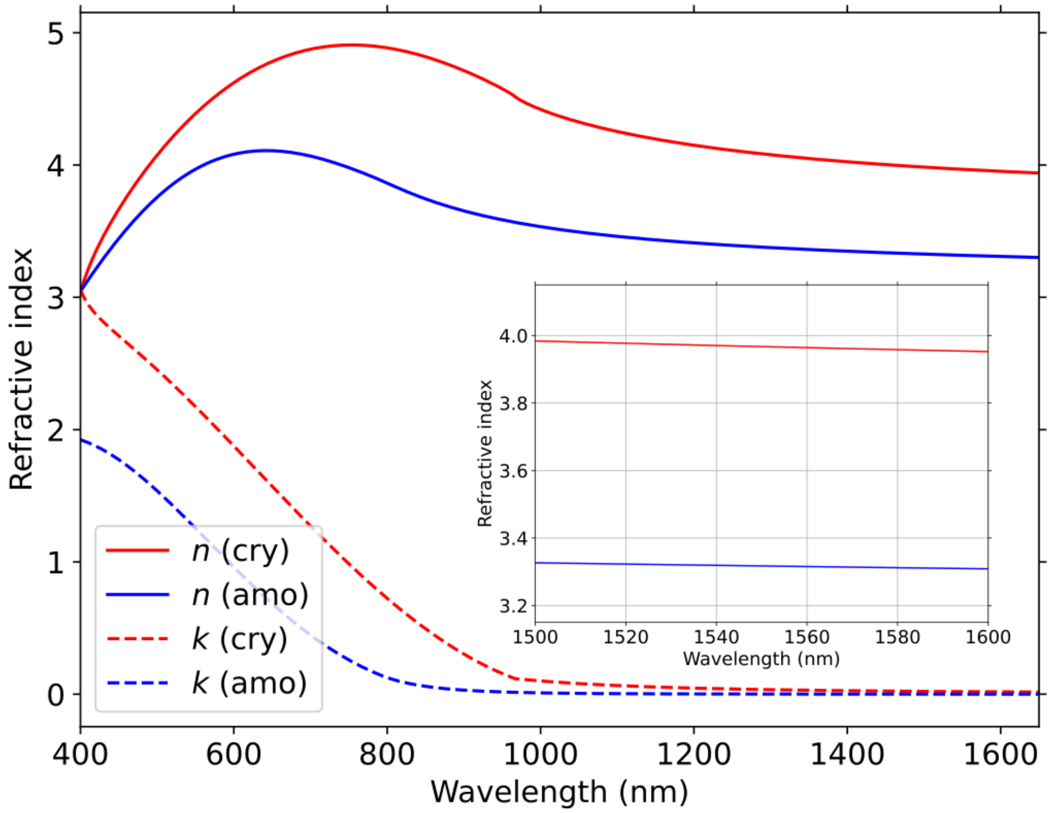

Figure S1. Ellipsometry measurements of a 180 nm thick thin-film of the PCM SbSe. The inset shows the wavelength range from 1500–1600 nm.

S2 Mode simulations of SbSe-covered waveguide

The values obtained from the ellipsometry measurements were used in optical simulations in Ansys Lumerical MODE. When PCM is deposited on top of an optical waveguide, the change in the refractive index of the PCM leads to a change in the effective refractive index, n_{eff}, of the hybrid waveguide-PCM system. Figure S2 shows cross-sectional mode profiles for the hybrid waveguide when the PCM is in the amorphous and crystalline state, respectively. In these simulations, the waveguide is a 500 nm wide rib waveguide with a 100 nm slab and the PCM is capped with 20 nm of ZnS-SiO_2.

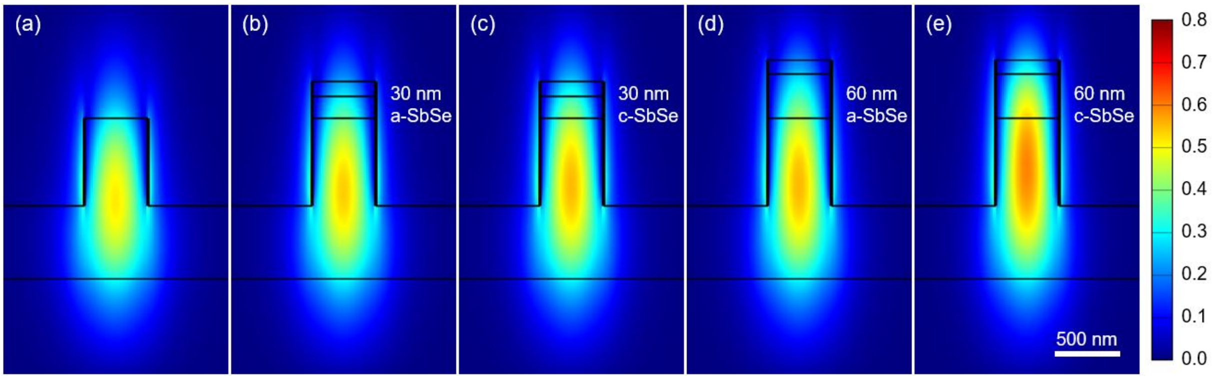

Figure S2. Cross-sectional mode profiles for the E_y field of the silicon-on-insulator waveguide (**a**) and hybrid waveguide with the PCM SbSe in the amorphous (**b, d**) and crystalline state (**c, e**) at $\lambda = 1550$ nm for PCM thickness 30 nm (**b, c**) and 60 nm (**d, e**). Note that the PCM is only on top of the waveguide in these simulations.

Table S2 summarizes the simulation results for an SbSe thickness of 30 nm and 60 nm on top of the waveguide. In the table, we have also included the length required for a full π phase shift, $L_\pi = \frac{\lambda}{2\Delta n_{\text{eff}}}$, for the wavelength $\lambda = 1550$ nm. As can be seen from the table, increasing the thickness of the PCM can significantly reduce the length of the device. However, an increased thickness will lead to increased losses.

Table S2: Results from MODE simulations for a hybrid waveguide, with the PCM SbSe only on top of the 500 nm wide waveguide with a 120 nm rib.

	30 nm	60 nm
$n_{\text{eff, cry}}$	2.6279	2.7312
$n_{\text{eff, amo}}$	2.5813	2.6319
$\Delta n_{\text{eff}} = n_{\text{eff, cry}} - n_{\text{eff, amo}}$	0.0466	0.0993
$L_\pi(\lambda = 1550\text{ nm})$	16.63 µm	7.81 µm

S3 Electrical characterization of silicon-on-insulator microheater

Figure S3 shows the *I–V* curves of the microheaters in this work, which we use to find the heater resistance $R \approx 1.5$ kΩ. It is worth noting that, similar to the heaters in the work by Ríos *et al.*, the devices show a roll-off effect at high voltages (≳6 V), due to the carrier mobility dependence on temperature (Fig. S3b) (*22*). Furthermore, as mentioned in Ríos *et al.* (*22*), the

function generator used in this work assumes a 50 Ω impedance at the microheater, but because the actual resistance is significantly higher, the voltage from the function generator is higher by a factor F, given as

$$F(R) = \frac{R}{R_{\text{load}}} \frac{R_{\text{load}} + R_{\text{int}}}{R + R_{\text{int}}}, \tag{S1}$$

where R is the actual resistance of the microheater, R_{load} = 50 Ω is the assumed resistance of the microheater, and R_{int} = 50 Ω is the internal resistance of the function generator.

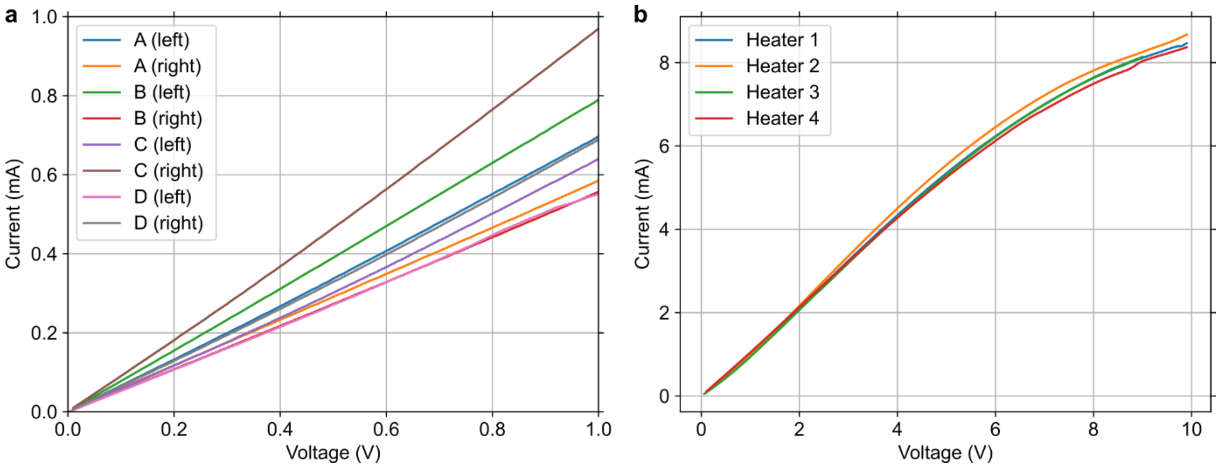

Figure S3: Current–voltage (*I*–*V*) sweeps of microheaters. **a**, *I*–*V* sweeps of the microheaters in the field-programmable coupler array unit cell in the main manuscript. **b**, Plots of heaters on a different device, showing saturation at large voltages.

S4 Detailed structural design parameters of phase shifter

A two-dimensional GDS design of the field-programmable coupler array (FPCA) unit cell in the main manuscript and one of its microheaters are shown in Supplementary Fig. S4**a,b**, respectively. The PCM and heater length are given in the main manuscript, while the rest of the parameters of the heaters are $w_{\text{clad}} = 3$ µm, $w_{\text{wg}} = 0.5$ µm, $w_{\text{PCM}} = 4.5$ µm, $L_2 = L_1 +$ 3.2 µm = 9.2 µm, $L_3 = 15$ µm, $w_1 = 2.5$ µm, $w_2 = 1$ µm, and $w_3 = 2.7$ µm.

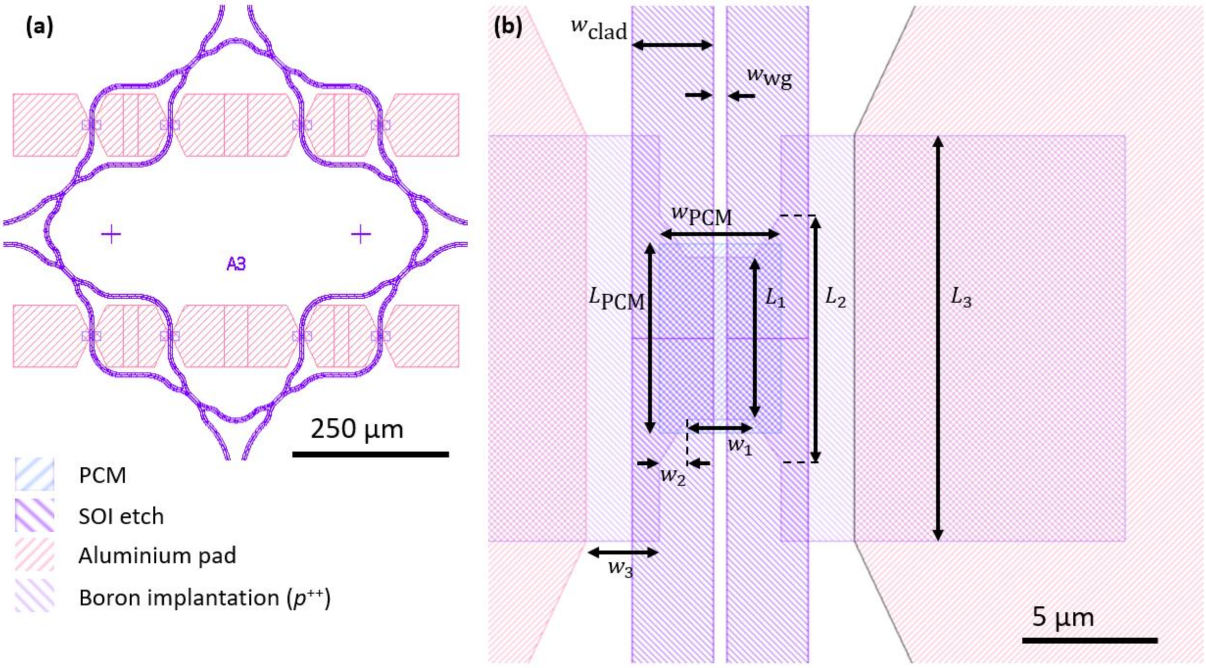

Figure S4: Two-dimensional GDS designs of the FPCA. **a**, Design of the field-programmable coupler array unit cell in the main manuscript. **b**, Microheater design with labelled structural parameters. The darkest purple corresponds to the half-etched silicon-on-insulator (SOI) waveguide slab (labelled SOI etch).

S5 Optical loss due to ion implantation

The loss attributed to the doping is 0.108 dB/µm, meaning that the total loss of the 7 µm heater is 0.76 dB, equivalent to 16 % of the power being lost. For a round trip with four tunable couplers, half of the light is lost to the doped silicon heaters.

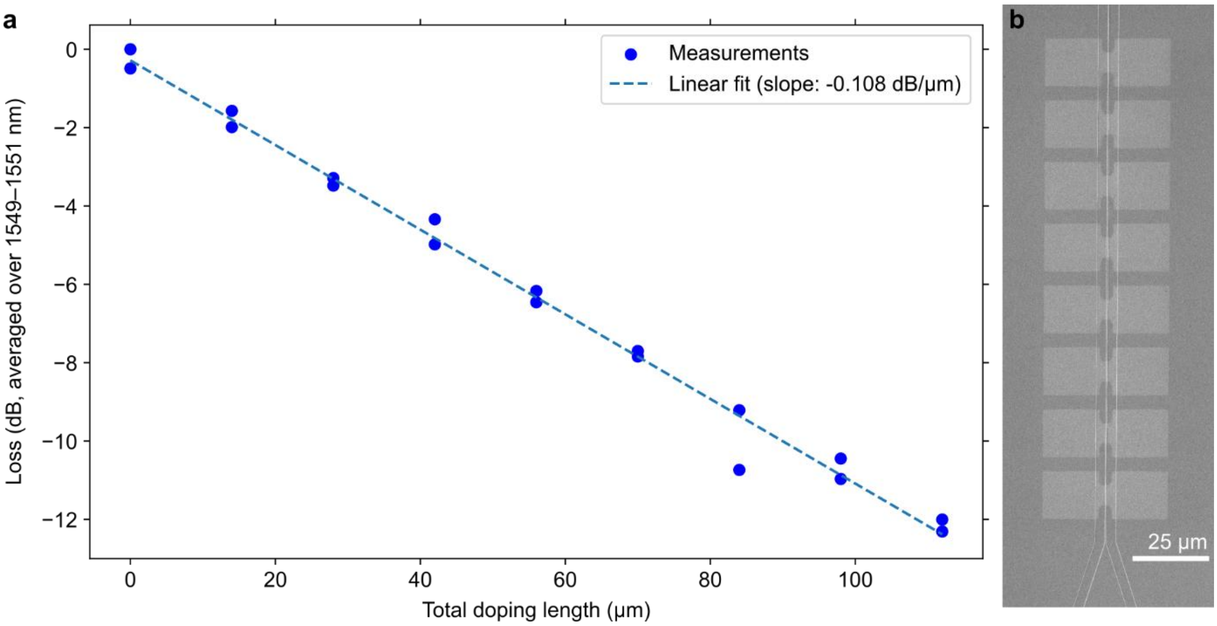

Figure S5: Optical loss measurement of microheaters. **a**, Optical loss due to ion implantation in the waveguides, averaged for transmission between 1549 and 1551 nm. **b**, Scanning electron micrograph of a waveguide section with several 7 µm long doped patches, used for the loss measurements.

S6 Optical measurements of SbSe on a rib-waveguide

To characterize the effective refractive index and loss of the hybrid waveguide, we fabricated two different structures: unbalanced MZIs with PCM on one arm and simple waveguides with PCM, shown in Supplementary Figures S6a,b, respectively. The results for the effective refractive index and loss are shown in Supplementary Figures S6c–f, for 30/60 nm of SbSe capped with 20 nm of ZnS-SiO_2. The two states of SbSe are amorphous (i.e., as-deposited) and crystalline (i.e., crystallized on a hotplate at 250 °C for 5 min).

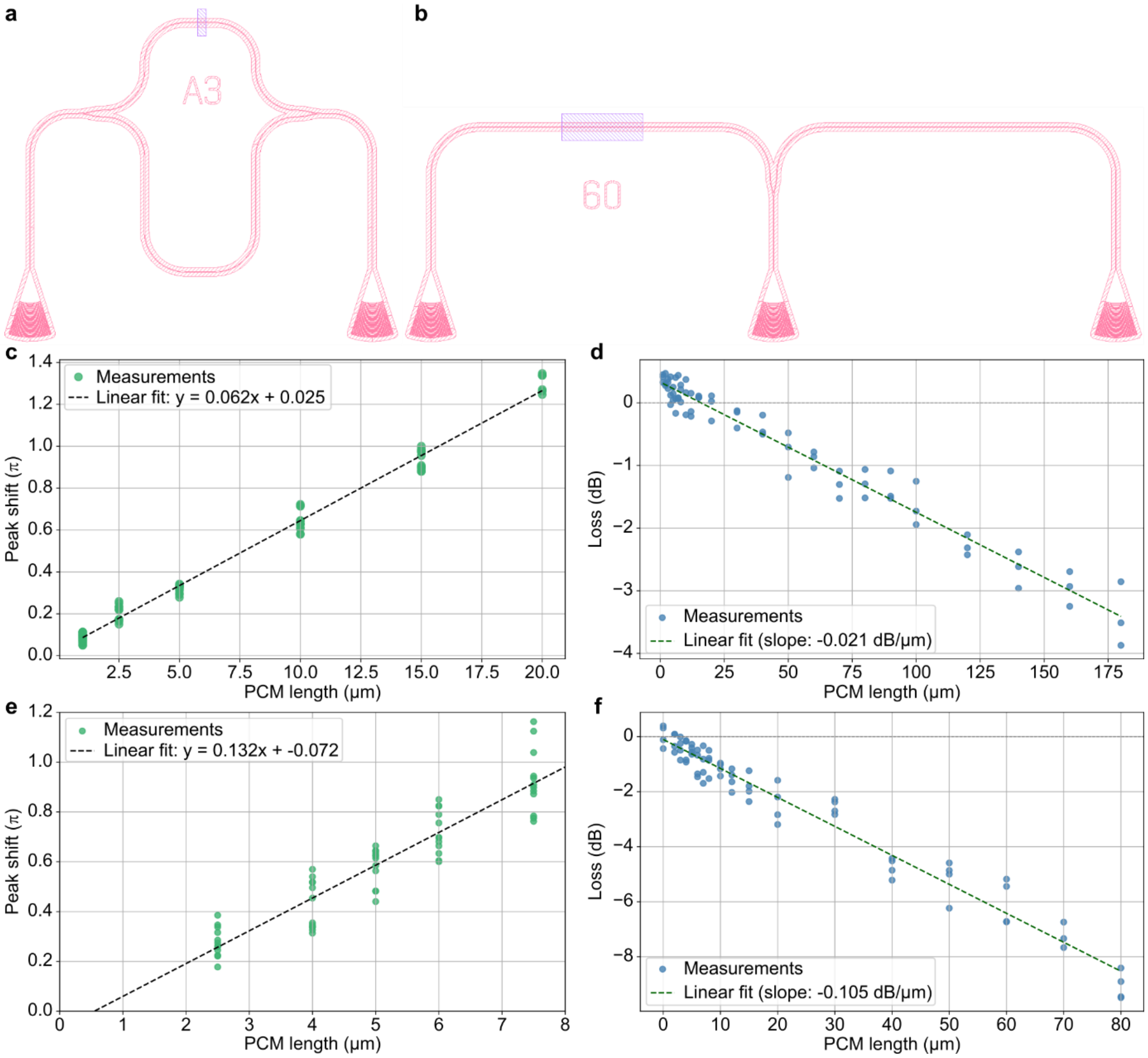

Figure S6: Optical measurements of hybrid waveguides with SbSe. **a**,**b**, The structures used for the measurements of effective refractive index (a) and loss (b). **c**,**e**, Phase shift (given in π, i.e., $\frac{\Delta\lambda}{\mathrm{FSR}/2}$ where FSR is the free spectral range) between the amorphous and crystalline states, as a function of the SbSe length along the waveguide, for 30 nm (c) and 60 nm (e) thick SbSe. **d**,**f**, Loss in dB of the crystalline state vs. the a bare waveguide as a function of the SbSe length along the waveguide, for 30 nm (d) and 60 nm (f) thick SbSe. There was no measurable increase in the loss of the hybrid waveguide with amorphous PCM vs. the bare waveguide.

The effective refractive index can be found using the formula

$$n_{\text{eff}} = \frac{\lambda}{L}\frac{\Delta\lambda}{\text{FSR}}, \qquad \text{(S2)}$$

where λ is the probe wavelength, L is the length of the PCM element along the waveguide, Δλ is the resonant wavelength shift, and FSR is the free spectral range, i.e., the distance between two resonances (*35*). The change of refractive index between the two states is found to be $\Delta n_{\text{eff}} = 0.0465$ for 30 nm SbSe, giving an $L_\pi = 16.1$ μm at $\lambda = 1550$ nm, which is in agreement with the $\Delta n_{\text{eff}} = 0.0466$ and $L_\pi = 16.6$ μm from simulations. For 60 nm SbSe, the same values are $\Delta n_{\text{eff}} = 0.103$ and $L_\pi = 7.6$ μm.

The loss of the crystalline state was measured to be 0.02 dB/μm for 30 nm SbSe and 0.11 dB/μm for 60 nm SbSe. This is in agreement with values from literature (*22*, *24*). The amorphous loss was immeasurable in our experimental setup.

S7 Fabrication steps

The fabrication steps are shown in Supplementary Figure S7. All fabrication steps were performed in our laboratories unless noted otherwise. The electron-beam lithography (EBL) system used for patterning was a JEOL JBX-5500FS with 50 kV acceleration voltage, waveguide etching was done in an Oxford Instruments PlasmaPro 80 Cobra inductively coupled plasma reactive-ion etching (ICP-RIE) system and ion implantation was done at the Surrey Ion Beam Centre at the University of Surrey. Annealing was performed in a Lenton Thermal Design LTF 12 tube furnace, metallization in a Plassys electron-beam evaporation

system, and radio-frequency (RF) sputtering in an AJA International ATC ORION 5 system.

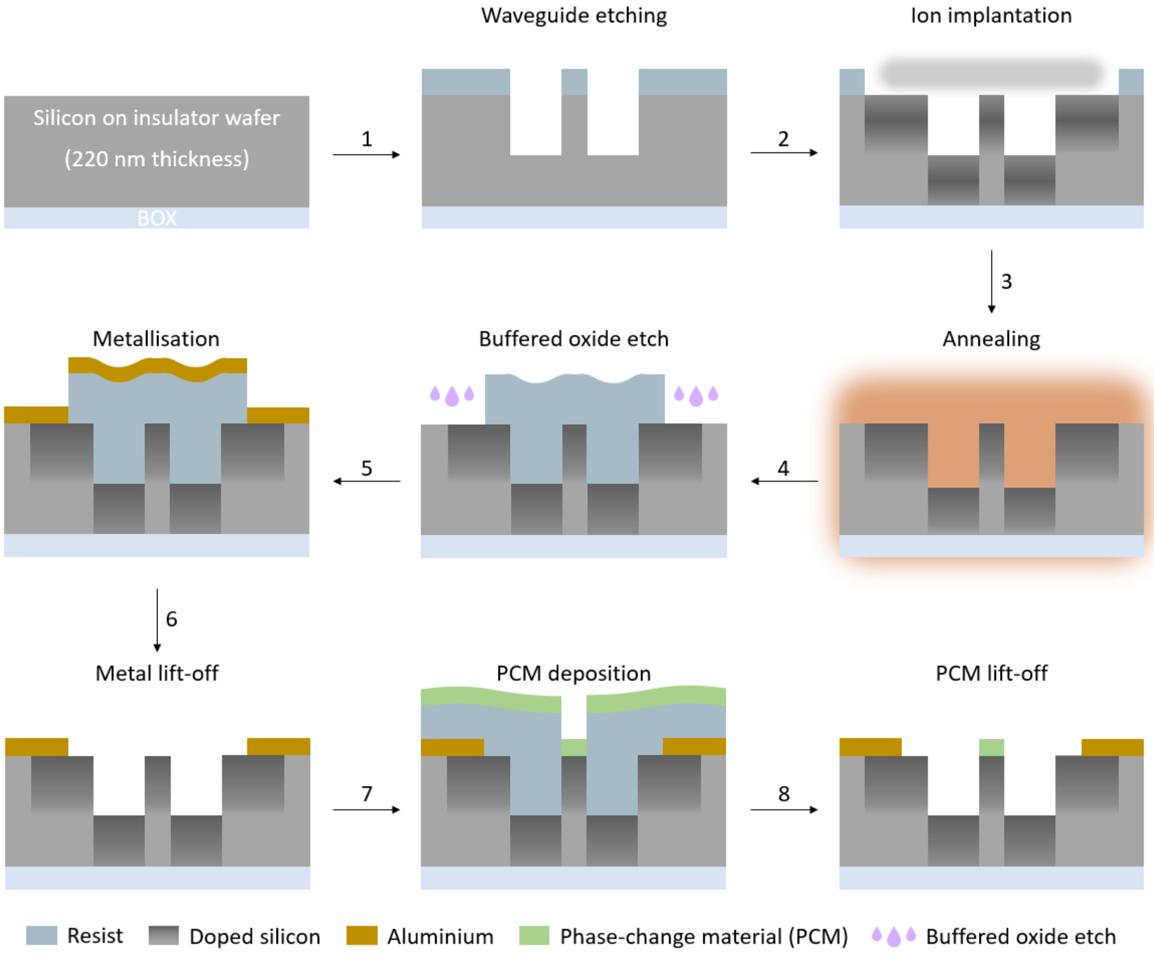

Figure S7: Steps to fabricate the doped silicon microheaters with phase-change material (PCM): (1) patterning with electron-beam lithography (EBL) and reactive-ion etching (RIE) etching of silicon on insulator (SOI) waveguides, (2) opening of windows with EBL and ion implantation, (3) tube furnace annealing for dopant activation, (4) opening of windows with EBL for metal deposition and buffered oxide etch of native oxide, (5) electron-beam evaporation of aluminium, (6) lift-off in acetone, (7) patterning with EBL and deposition of PCM (Sb_2Se_3) and capping layer (ZnS-SiO_2, not shown) on the doped waveguides, and finally (8) lift-off in acetone. Not shown in the figure are spin-coating before step 1, 4, and 7; resist removal and spin-coating before step 2; and resist removal and piranha clean before step 3.

S8 Optical loss of SbSe-based tunable coupler

The spectra in this work are normalized to the transmission spectrum of a plain waveguide, to remove the Gaussian-like envelope of the spectra caused by the grating couplers. The tunable coupler we measured in the main manuscript has an insertion loss (IL) relative to the simple waveguide of IL_{cross} = -3.2 dB and IL_{bar} = -4.0 dB for λ = 1582.5 nm, of which an estimated 0.8 dB stem from the ion implants and at least 0.1 dB from each directional coupler (*20, 37*). The remaining loss is due to the PCM and the longer optical path length of the waveguides of the MZI.

It is challenging to estimate exactly the insertion loss of the devices, due to the variation in grating couplers, the fibres of the fibre array, alignment, etc. Furthermore, because of limited chip space, we did not have many reference devices for loss measurements. In the figures shown in this paper, we have normalized the transmission to that of a plain waveguide and accounted for the slight difference in performance of the photodetectors used. One issue with the way the measurements are conducted is that the normalized transmission can appear tilted (as seen in Fig. 2 and Fig. 3) if the Gaussian-like shape of the transmission is slightly offset to that of the reference measurement, which makes it hard to estimate the loss of our devices. Furthermore, the different fibres of the fibre array used has slightly different transmission.

For the FPCA unit cell, the total system loss relative to a plain waveguide was -4.5 dB, -8.1 dB, -8.7 dB, and -9.3 dB at $\lambda = 1584$ nm for the configurations in Fig. 3(b-e), respectively, which shows that from our measurements the loss of the devices is not necessarily linear. Further, the loss of the delay line in Fig. 4(b) was -11.0 dB at the same wavelength.

Because the measurement parameters (waveguide length, grating couplers, optical fibres, and photodetector) are the same for the measurements in Fig. 3(d) and the delay line in Fig. 4(b), we can estimate the loss of our devices from these measurements. In the former case, we have coupler A in the bar state and in the latter coupler A is in the cross state with the three other

couplers in the bar state, and with two passes through coupler A. The loss of coupler A is $IL_{NW,\ bar} - x = -4.5$ dB and $2IL_{NW,\ cross} - x = -11.0\ \text{dB} - 3 \times IL_{bar}$, where x is extra loss and $IL_{bar/cross}$ is the insertion loss of the bar/cross state couplers. Assuming $IL_{bar} = IL_{cross} - 0.4$ dB due to the difference in PCM loss (because one arm is crystalline and the other amorphous), we get $IL_{bar} = -1.83$ dB and $IL_{cross} = -1.43$ dB.

S9 Switching behaviour

Here, we show experimental switching measurements from individual MZIs, supplementing the multi-level data in Fig. 2F in the main manuscript. Table S3 summarizes the switching parameters used for the amorphization pulses in Fig. 2F.

Table S3: Overview of switching parameters used for complete amorphization for the device shown in Fig. 2F.

t (ns)	*V* (V)	# of times
375	14.9	10
375	15.2	3
395	15.2	3
400	15.4	10
425	16.4	10
425	17.0	10
435	17.2	11
435	16.8	3

In Fig. S8(a), we show the change in transmission from the bar port of an MZI coupler under sequential crystallization of one of the phase shifters, with the phase shifter of interest initially set to the amorphous state and the other phase shifter in the crystalline state. The plots show

that we are able to crystallize ~50 % of the PCM over several intermediate steps, with switching parameters shown in Table S4.

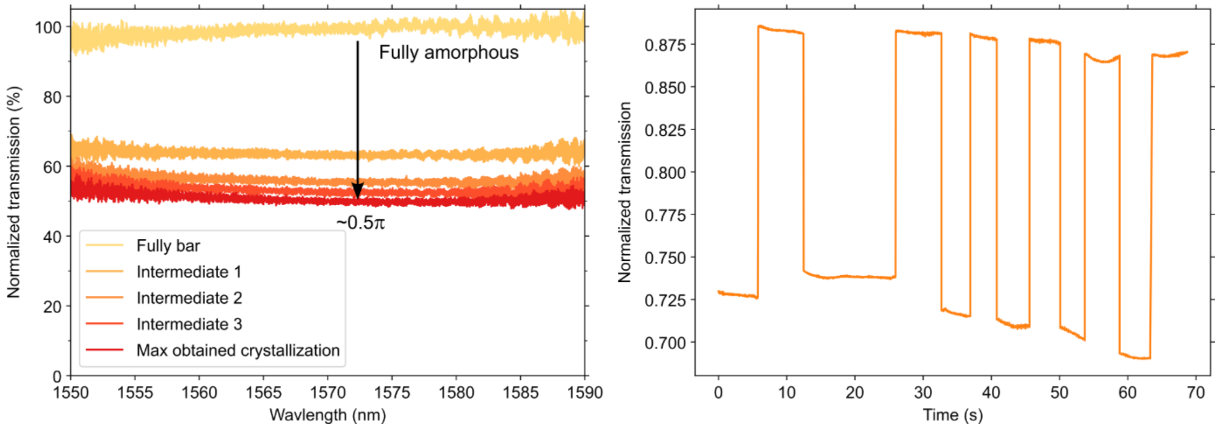

Figure S8: Switching behaviour of couplers. **a**, Normalized transmission spectra from the bar port of an individual MZI coupler with the phase shifter of interest set to the amorphous state and the other phase shifter in the crystalline state. The plots show that we are able to crystallize ~50 % of the PCM over several intermediate steps, with pulse parameters shown in Table S4. **b**, Repeated amorphous (600 ns, 18.7 V) and crystalline pulses (cry: 200 μs, 7.4 V), switching ~13 % of the PCM volume.

Table S4: Overview of switching parameters used in Fig. S8(a), for crystallization of ~50 % of the PCM volume. The time durations t_1, t_2, and t_3 refer to the parameters in Eq. S5.

Event	t_1 (μs)	t_2 (μs)	t_3 (μs)	V (V)	# of times
Bar ⟶ Int1	300	700	1200	7.1	6
Int1 ⟶ Int2	300	700	1200	7.5	1
	300	700	1700	7.5	1
	300	700	1700	7.7	1
Int2 ⟶ Int3	300	700	1700	8.1	1
	400	600	1700	7.5	1

	400	600	1700	8.1	1
Int3 → Max crystallization	500	500	1700	8.3	1
	450	550	1700	8.5	1
	500	500	1700	8.5	1
	500	500	1700	8.7	1

Figures S8(b) shows cyclability experiments, where we measure the transmission of an MZI and switch one of the phase shifters repeatedly. To calculate the switched volume of the PCM, ΔV, we need to account for the sinusoidal shape of the MZI transmission and therefore use the equation

$$\Delta V = \frac{\Delta\varphi}{\pi} = \frac{2\arccos\sqrt{T_1} - 2\arccos\sqrt{T_0}}{\pi}, \tag{S3}$$

where T_1 and T_0 are the normalized transmission levels and we assume that the entire PCM volume gives a π shift. In Fig. S8(b), we are able to show repeated switching equivalent of ~13 % of the PCM.

To amorphize the PCM patch, we sent rectangular pulses of energy, *E*, given as:

$$E_{\text{rectangular}} = Pt = \frac{V^2}{R}t, \tag{S4}$$

where *P* is the power consumed, *t* is the duration of the electrical pulse, and *V* and *R* are, respectively, the voltage applied to and the resistance of the microheater. To completely amorphize the PCM patch, we sent pulses with a total energy of 1.2 µJ (individual pulse parameters given in Table S5). Note, however, that the first four pulses alone change the transmission from 100 % to 6.5 %.

Table S5: Overview of switching parameters and transmission levels used for complete amorphization for the device with the smallest switching energy quoted in the main manuscript.

t (ns)	*V* (V)	# of times	Transmission level after pulse (%)
400	15.2	1	93
425	16.0	1	74
450	16.5	1	43
500	16.7	10	6.5, 4.3, 3.1, 2.1, 1.4, 1.0, 0.75, 0.60, 0.42, 0.28

For crystallization, we also sent several trapezoidal pulses with energy given by

$$E_{\text{trapezoidal}} = \frac{V_1^2}{R}\left(t_1 + \int_0^{t_2}\left(1 - \frac{t}{t_3}\right)dt\right). \qquad \text{(S5)}$$

As shown in Fig. S8(a), we were only able to obtain a total crystallization of 50 %, with 15 individual pulses with energies of up to 45 μJ and a total energy of 583 μJ (Table S4). In this work, we estimate the total energy for full crystallization to be twice of that, i.e., 1.2 mJ. It is worth pointing out that in these calculations, we have accounted for the voltage factor *F*, but not the saturation effect in Fig. S3(b). If we assume that the saturation was similar to that in Fig. S3(b), at 8.5 V the energy is 6 % lower and above 10 V at least 12 % lower than when using the resistance value obtained at 1 V.

S10 Synthesized ring resonator behaviour

Figure S9 shows the behaviour of the synthesized ring resonator. Figures a-c show zoomed-in regions with fits to the resonances. We use the data analysis software Origin to fit nine resonances and show here seven of the fits and the cumulative fit for the synthesized ring with the input coupler in the bar state (Fig. S9(a)), near critical coupling (Fig. S9(b)), and in the

cross state (Fig. S9(c)), corresponding to the plots in Fig. 4(b). We were not able to fit the curve for the wavelength region around 1584 nm for the bar state plot in Fig. S9(c).

Furthermore, we show simulation data from the simulation software Ansys Lumerical Interconnect. In Fig. S9(d), we show the setup used in the software. Note that there are actually three yellow MZI couplers (the MZIs forming the ring), but two have been removed to save space. In Fig. S9(e), we show a measurement from a wider section of the plot in Fig. S9(c) on a dB scale, normalized to the input of a plain waveguide. The shape of the envelope is determined by the wavelength dependence on the coupling condition of the directional couplers. In Fig. S9(f), we show simulation data from Interconnect for the following parameters: the cyan MZI is close to the cross state but has a tiny phase difference between the arms of 1.38°, the yellow MZIs are in the bar state, the attenuation of each arm of the MZI is 0.49 dB, the total circumference of the synthesized ring is 1935.3 µm, and the directional couplers used for coupling into and out of the MZIs have a wavelength dependence given by $C = 4.5 \times 10^{-3} \lambda - 6.6298$, which gives 50 % coupling at 1584.4 nm and ±10 % coupling at ±22.2 nm. The simulation data shows good overlap with the experimental measurement.

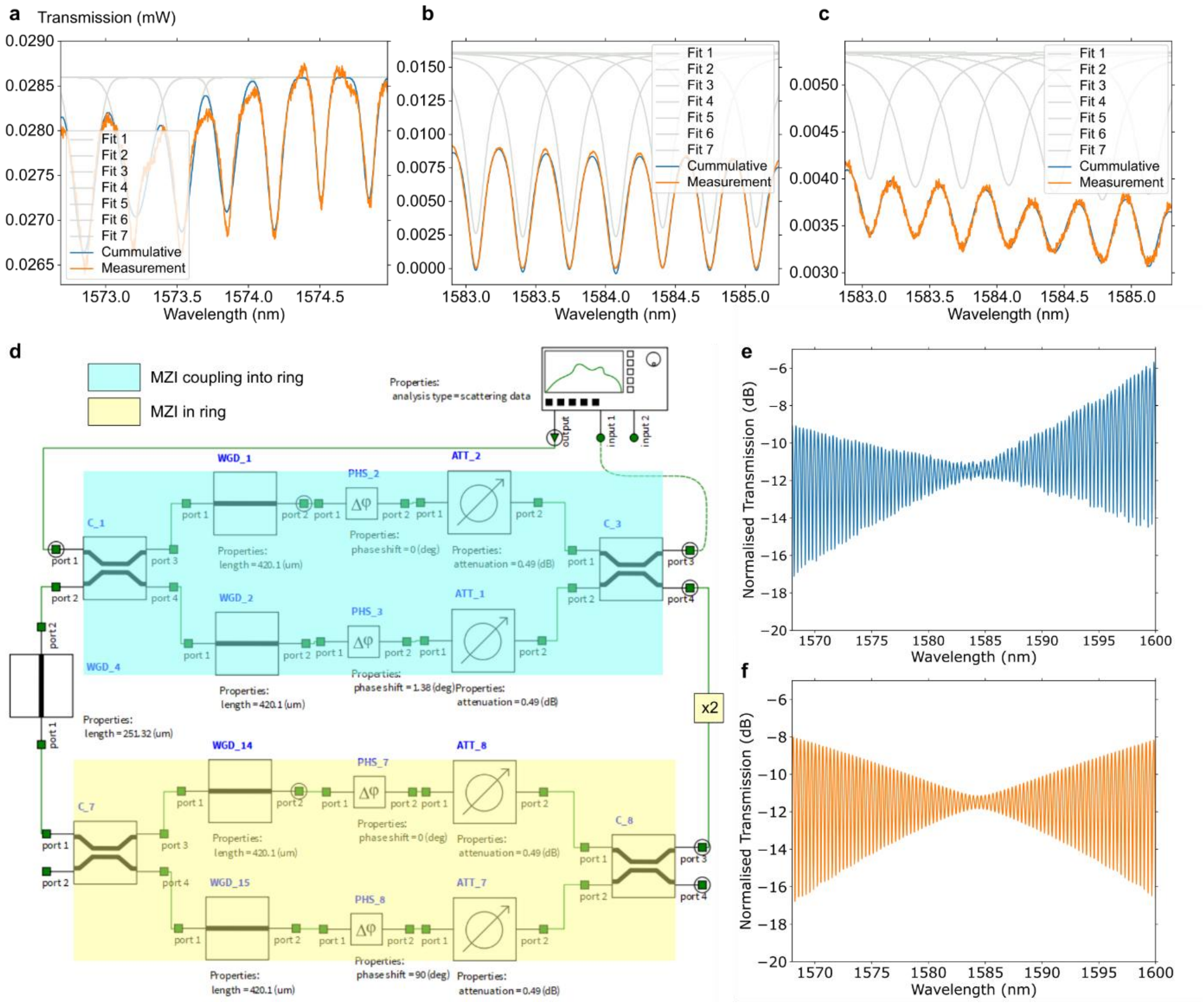

Figure S9: Behaviour of the synthesized ring resonator. **a–c**, Lorentzian fits to the resonances of the synthesized ring resonator when the in-coupler is in the bar state (**a**), near critical coupling (**b**), and in the cross state (**c**), corresponding to the plots in Fig. 4(b). The fit indices increase from left to right, with the cumulative fit being based on nine resonances (one left and one right of the region). **d**, Setup used in the simulation software Lumerical INTERCONNECT. The cyan rectangle indicates the MZI coupling into the ring, whereas the yellow indicates one of the MZIs forming the ring. Note that there are three yellow MZI couplers, but two have been removed to save space. **e**, Normalized transmission spectrum of the measurement in Fig. S9(c). The shape of the envelope is determined by the wavelength dependence on the coupling condition of the directional couplers. **f**, Normalized simulation data from INTERCONNECT with imperfect directional couplers.

S11 Future projections for footprint

The miniaturization of the MZI couplers in this work is restricted by the size of the electrical pads, in particular the pad inside the MZI, because we need to make contact to the pads with RF probes. If we could instead use electrical vias, for a future design, the optical path length of the PCM-based tunable coupler could be less than 100 µm. Furthermore, if the MZI is set in a push-pull configuration, we only need $\frac{\pi}{2}$ per arm to set the coupling, which means the PCM patches can be shorter. For the length calculation, we assumed moderate values for the bend radius (r = 10 µm), the bend angle between the directional couplers and MZI arms (36° = $\pi/5$), and the length of the coupling section of the directional coupler to achieve 50/50 coupling (L_{DC} = 8.9 µm). The length of the straight section of the MZI (L_{PCM}) is set to 25.0 µm, equivalent of $3\pi/2$ phase shift for 30 nm thick SbSe, which in a push-pull configuration leaves a π phase shift for phase control, which is sufficient for most applications. Alternatively, we can extend the length of the PCMs to 41.8 µm to give 2π phase control, or even 50.1 µm (3π) for coupling (π) and phase (2π) control without the push-pull configuration.

The total optical path length is thus given by

$$L_{\mathrm{total}} = 2L_{\mathrm{DC}} + 4r\frac{\pi}{5} + 2r\frac{\pi}{n} + L_{\mathrm{PCM}} = 53.4 + L_{\mathrm{PCM}}, \tag{S6}$$

where n is the number of MZIs per unit cell. The total optical path length is therefore in the range from 78.4 µm (for 25 µm long PCM in hexagonal lattice with push-pull) to 108.7 µm (for 50.1 µm long PCM in square lattice without push-pull). With these phase shifter lengths, the total loss of the PCM is between 0.5 dB and 1.0 dB.

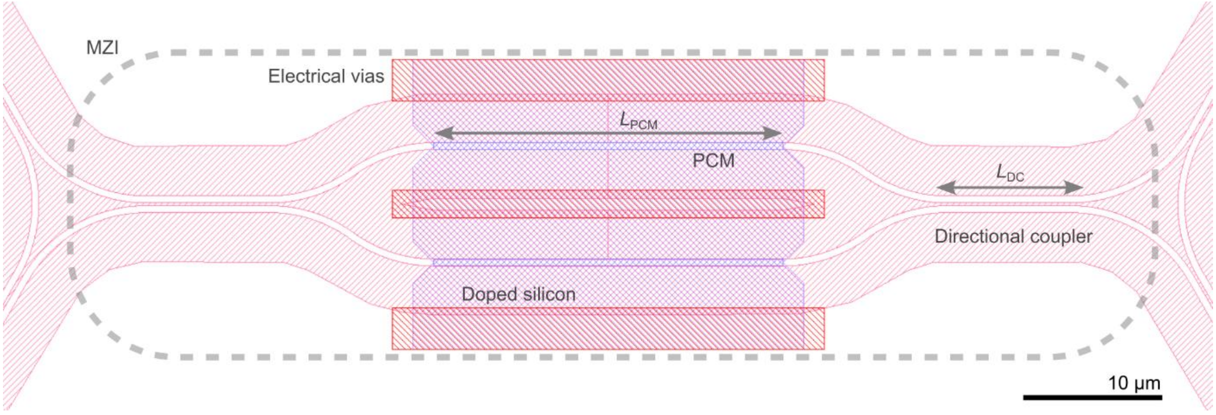

Figure S10: GDS design of a future MZI coupler. Note how the input and output bends from the directional couplers are part of the bend between MZI couplers.

REFERENCES

1. D. Marpaung, J. Yao, J. Capmany, Integrated microwave photonics. *Nat. Photonics* **13**, 80–90 (2019).

2. P. Ghelfi, F. Laghezza, F. Scotti, G. Serafino, A. Capria, S. Pinna, D. Onori, C. Porzi, M. Scaffardi, A. Malacarne, V. Vercesi, E. Lazzeri, F. Berizzi, A. Bogoni, A fully photonics-based coherent radar system. *Nature* **507**, 341–345 (2014).

3. X. Zhou, D. Yi, D. W. U. Chan, H. K. Tsang, Silicon photonics for high-speed communications and photonic signal processing. *Npj Nanophoton.* **1**, 1–14 (2024).

4. J. Feldmann, N. Youngblood, M. Karpov, H. Gehring, X. Li, M. Stappers, M. Le Gallo, X. Fu, A. Lukashchuk, A. S. Raja, J. Liu, C. D. Wright, A. Sebastian, T. J. Kippenberg, W. H. P. Pernice, H. Bhaskaran, Parallel convolutional processing using an integrated photonic tensor core. *Nature* **589**, 52–58 (2021).

5. R. Yin, H. Xiao, Y. Jiang, X. Han, P. Zhang, L. Chen, X. Zhou, M. Yuan, G. Ren, A. Mitchell, Y. Tian, Integrated WDM-compatible optical mode division multiplexing neural network accelerator. *Optica* **10**, 1709–1718 (2023).

6. Y. Shen, N. C. Harris, S. Skirlo, M. Prabhu, T. Baehr-Jones, M. Hochberg, X. Sun, S. Zhao, H. Larochelle, D. Englund, M. Soljačić, Deep learning with coherent nanophotonic circuits. *Nat. Photonics* **11**, 441–446 (2017).

7. S. Gyger, J. Zichi, L. Schweickert, A. W. Elshaari, S. Steinhauer, S. F. Covre da Silva, A. Rastelli, V. Zwiller, K. D. Jöns, C. Errando-Herranz, Reconfigurable photonics with on-chip single-photon detectors. *Nat. Commun.* **12**, 1408 (2021).

8. C. Sun, M. Georgas, J. Orcutt, B. Moss, Y.-H. Chen, J. Shainline, M. Wade, K. Mehta, K. Nammari, E. Timurdogan, D. Miller, O. Tehar-Zahav, Z. Sternberg, J. Leu, J. Chong, R. Bafrali, G. Sandhu, M. Watts, R. Meade, M. Popović, R. Ram, V. Stojanović, A monolithically-integrated chip-to-chip optical link in bulk CMOS. *IEEE J. Solid-State Circuits* **50**, 828–844 (2015).

9. D. Pérez-López, A. Gutierrez, D. Sánchez, A. López-Hernández, M. Gutierrez, E. Sánchez-Gomáriz, J. Fernández, A. Cruz, A. Quirós, Z. Xie, J. Benitez, N. Bekesi, A. Santomé, D. Pérez-Galacho, P. DasMahapatra, A. Macho, J. Capmany, General-purpose programmable photonic processor for advanced radiofrequency applications. *Nat. Commun.* **15**, 1563 (2024).

10. N. Farmakidis, H. Yu, J. S. Lee, J. Feldmann, M. Wang, Y. He, S. Aggarwal, B. Dong, W. H. P. Pernice, H. Bhaskaran, Scalable high-precision trimming of photonic resonances by polymer exposure to energetic beams. *Nano Lett.* **23**, 4800–4806 (2023).

11. A. Varri, S. Taheriniya, F. Brückerhoff-Plückelmann, I. Bente, N. Farmakidis, D. Bernhardt, H. Rösner, M. Kruth, A. Nadzeyka, T. Richter, C. D. Wright, H. Bhaskaran, G. Wilde, W. H. P. Pernice, Scalable non-volatile tuning of photonic computational memories by automated silicon ion implantation. *Adv. Mater.* **36**, e2310596 (2024).

12. L. Zhuang, C. G. H. Roeloffzen, M. Hoekman, K.-J. Boller, A. J. Lowery, Programmable photonic signal processor chip for radiofrequency applications. *Optica* **2**, 854–859 (2015).

13. D. Pérez, I. Gasulla, L. Crudgington, D. J. Thomson, A. Z. Khokhar, K. Li, W. Cao, G. Z. Mashanovich, J. Capmany, Multipurpose silicon photonics signal processor core. *Nat. Commun.* **8**, 636 (2017).

14. L. Lu, S. Zhao, L. Zhou, D. Li, Z. Li, M. Wang, X. Li, J. Chen, 16×16 non-blocking silicon optical switch based on electro-optic Mach-Zehnder interferometers. *Opt. Express* **24**, 9295–9307 (2016).

15. W. Liu, M. Li, R. S. Guzzon, E. J. Norberg, J. S. Parker, M. Lu, L. A. Coldren, J. Yao, A fully reconfigurable photonic integrated signal processor. *Nat. Photonics* **10**, 190–195 (2016).

16. W. Bogaerts, D. Pérez, J. Capmany, D. A. B. Miller, J. Poon, D. Englund, F. Morichetti, A. Melloni, Programmable photonic circuits. *Nature* **586**, 207–216 (2020).

17. D. Pérez-López, A. M. Gutierrez, E. Sánchez, P. DasMahapatra, J. Capmany, Integrated photonic tunable basic units using dual-drive directional couplers. *Opt. Express* **27**, 38071–38086 (2019).

18. D. Pérez-López, A. Gutiérrez, J. Capmany, Silicon nitride programmable photonic processor with folded heaters. *Opt. Express* **29**, 9043–9059 (2021).

19. M. Dong, G. Clark, A. J. Leenheer, M. Zimmermann, D. Dominguez, A. J. Menssen, D. Heim, G. Gilbert, D. Englund, M. Eichenfield, High-speed programmable photonic circuits in a cryogenically compatible, visible–near-infrared 200 mm CMOS architecture. *Nat. Photonics* **16**, 59–65 (2022).

20. D. U. Kim, Y. J. Park, D. Y. Kim, Y. Jeong, M. G. Lim, M. S. Hong, M. J. Her, Y. Rah, D. J. Choi, S. Han, K. Yu, Programmable photonic arrays based on microelectromechanical elements with femtowatt-level standby power consumption. *Nat. Photonics* **17**, 1089–1096 (2023).

21. M. Delaney, I. Zeimpekis, D. Lawson, D. W. Hewak, O. L. Muskens, A new family of ultralow loss reversible phase-change materials for photonic integrated circuits: Sb_2S_3 and Sb_2Se_3. *Adv. Funct. Mater.* **30**, 2002447 (2020).

22. C. Ríos, Q. Du, Y. Zhang, C.-C. Popescu, M. Y. Shalaginov, P. Miller, C. Roberts, M. Kang, K. A. Richardson, T. Gu, S. A. Vitale, J. Hu, Ultra-compact nonvolatile phase shifter based on electrically reprogrammable transparent phase change materials. *PhotoniX* **3**, 26 (2022).

23. R. Chen, A. Tang, J. Dutta, V. Tara, J. Ye, Z. Fang, A. Majumdar, NEO-PGA: Nonvolatile electro-optically programmable gate array. arXiv:2506.18592 (2025). https://doi.org/10.48550/arXiv.2506.18592.

24. X. Yang, L. Lu, Y. Li, Y. Wu, Z. Li, J. Chen, L. Zhou, Non-volatile optical switch element enabled by low-loss phase change material. *Adv. Funct. Mater.* **33**, 2304601 (2023).

25. W. Zhou, B. Dong, N. Farmakidis, X. Li, N. Youngblood, K. Huang, Y. He, C. David Wright, W. H. P. Pernice, H. Bhaskaran, In-memory photonic dot-product engine with electrically programmable weight banks. *Nat. Commun.* **14**, 2887 (2023).

26. C. Lian, Y.-S. Huang, H. Sun, H. Yu, C.-C. Popescu, T. Gu, S. A. Vitale, I. Takeuchi, J. Hu, C. Ríos, "Phase and amplitude trimming of photonic integrated circuits using phase change materials" in *2022 IEEE Photonics Society Summer Topicals Meeting Series (SUM)* (IEEE, 2022), pp. 1–2; https://ieeexplore.ieee.org/document/9858196.

27. Y.-S. Huang, C.-Y. Lee, I. Takeuchi, C. A. R. Ocampo, Optical phase change materials. *Annu. Rev. Mat. Res.* **55**, 255–283 (2025).

28. G. W. Burr, M. J. Breitwisch, M. Franceschini, D. Garetto, K. Gopalakrishnan, B. Jackson, B. Kurdi, C. Lam, L. A. Lastras, A. Padilla, B. Rajendran, S. Raoux, R. S. Shenoy, Phase change memory technology. *J. Vac. Sci. Technol. B.* **28**, 223–262 (2010).

29. I. B. Sharuddin, L. Lee, "An ultra-low power and area efficient 10 bit digital to analog converter architecture" in *2014 IEEE International Conference on Semiconductor Electronics (ICSE2014)* (IEEE, 2014), pp. 305–308; https://ieeexplore.ieee.org/document/6920858/.

30. N. A. B. A. Taib, M. Mamun, L. F. Rahman, F. H. Hashim, A low power op amp for 3-bit digital to analog converter in 0.18 µm CMOS process. *RJASET* **5**, 2592–2598 (2013).

31.8 Channel, 12-/10-/8-Bit, 2.7-V To 5.5-V Low Power Digital-to-Analog Converters with Power Down and Internal Reference (Texas Instruments, 2008); https://www.ti.com/lit/ds/symlink/tlv5632.pdf?ts=1754894385154.

32.10 Bit, Low-Power, Quad, Voltage-Output DAC with Serial Interface (Maxim Integrated Products, 2008); https://www.mouser.co.uk/datasheet/2/609/MAX5741-3470420.pdf.

33. H. Fan, D. Li, K. Zhang, Y. Cen, Q. Feng, F. Qiao, H. Heidari, A 4-channel 12-bit high-voltage radiation-hardened digital-to-analog converter for low orbit satellite applications. *IEEE Trans. Circuits Syst. I Regul. Pap.* **65**, 3698–3706 (2018).

34. H. Zhang, L. Zhou, B. M. A. Rahman, X. Wu, L. Lu, Y. Xu, Ultracompact Si-GST hybrid waveguides for nonvolatile light wave manipulation. *IEEE Photonics J.* **10**, 1–10 (2018).

35. A. H. El-Saeed, A. Elshazly, H. Kobbi, R. Magdziak, G. Lepage, C. Marchese, Low-loss silicon directional coupler with arbitrary coupling ratios for broadband wavelength operation based on bent waveguides. *J. Lightwave Technol.* **42**, 6011–6018 (2024).
